# Efficacy of Cryotherapy After Inferior Alveolar Nerve Block in Symptomatic Irreversible Pulpitis: A Systematic Review and Meta-Analysis of Randomized Controlled Trials

**DOI:** 10.7759/cureus.84046

**Published:** 2025-05-13

**Authors:** Abdulwahab T Alenezi, Mohammed Alkandari, Meshari Alkandari, Rashed Alhallaq, Abdullah Alobaidan, Mubarak Alobaidan, Mohammad Almutairi, Mubarak Alajmi, Yousef Alajmi, Sayed A Alsaleh

**Affiliations:** 1 Department of Dentistry, Saad Al-Abdullah Health Center Block 2, Al Jahra, KWT; 2 Department of Dentistry, Rumaithiya Polyclinic, Kuwait City, KWT; 3 Department of Dentistry, West Salmiya Polyclinic, Kuwait City, KWT; 4 Department of Dentistry, Al Adan Specialized Health Center, Kuwait City, KWT; 5 Department of Dentistry, Sulaibyia Polyclinic, Al Jahra, KWT; 6 Department of Oral and Maxillofacial Surgery, Al Jahra Hospital, Al Jahra, KWT; 7 Department of Dentistry, Oyoun Polyclinic, Al Jahra, KWT; 8 Department of Dentistry, West Hawally Polyclinic, Hawally, KWT

**Keywords:** meta-analysis, modified dental anxiety scale, oral cryotherapy, pain, symptomatic irreversible pulpitis

## Abstract

The inferior alveolar nerve block (IANB) is routinely employed to achieve pulpal anesthesia during endodontic procedures. Cryotherapy is a well-established method for managing postoperative pain; however, evidence regarding its efficacy in enhancing IANB outcomes remains limited. This systematic review and meta-analysis aimed to evaluate the efficacy of cryotherapy application following IANB in patients with symptomatic irreversible pulpitis. A comprehensive search was conducted in PubMed, Scopus, Web of Science (WOS), and Cochrane Central from inception to February 2025 for randomized controlled trials (RCTs) assessing cryotherapy application after IANB. The primary outcomes included success rates, pain intensity measured using the Visual Analogue Scale (VAS), and anxiety levels assessed by the Dental Anxiety Scale-Revised (DAS-R). Dichotomous data were analyzed using odds ratios (ORs) with 95% confidence intervals (CIs), while continuous data were analyzed using mean differences (MDs) with 95% CIs under a random-effects model. All statistical analyses were performed using STATA version 18MP (www.stata.com). Six RCTs involving 506 patients were included. Ice pack application following IANB significantly improved success rates compared to no cryotherapy (OR: 1.44; 95% CI: 1.04-2.00; p = 0.03), whereas no significant difference was observed with Endo Ice. Cryotherapy also resulted in significantly greater pain reduction intraoperatively and at one hour postoperatively compared to control (MD = -0.83; 95% CI: -1.61 to -0.05; p = 0.04 and MD = -3.08; 95% CI: -5.56 to -0.60; p = 0.01, respectively). In conclusion, cryotherapy application following IANB enhances anesthetic efficacy and reduces pain in patients with irreversible pulpitis. Further high-quality trials with longer follow-up durations are recommended.

## Introduction and background

Symptomatic irreversible pulpitis (SIP) is a painful inflammatory condition of the dental pulp that ultimately progresses to necrosis, necessitating endodontic intervention [1]. Patients with SIP typically present with either spontaneous pain or pain induced by stimuli such as percussion or thermal challenges. The pain often persists for more than 30 seconds following the removal of the stimulus, underscoring the importance of achieving effective pulpal anesthesia to ensure successful endodontic treatment [1].

The mandibular first molar, as the earliest permanent tooth to erupt, is particularly prone to carious lesions and represents the most frequently treated tooth in endodontics [2]. The inferior alveolar nerve block (IANB) is the most commonly employed technique for achieving pulpal anesthesia in the mandibular quadrant [3]. However, its efficacy in SIP patients remains suboptimal, with reported success rates ranging from 10% to 75% in the general population [4,5] and 15% to 57% specifically in patients with SIP [6]. These findings highlight the clinical need for adjunctive strategies to improve the anesthetic success of IANB, especially in patients with inflamed pulps. Various techniques have been investigated to address this limitation, including pre-anesthetic medications, supplemental injections, and alternative mandibular block methods [7].

Cryotherapy, a technique of ice application, has been widely used in both acute and chronic pain management due to its ability to reduce tissue temperature and thus induce vasoconstriction and reduce local tissue metabolism, inflammatory mediators, and neuropeptides, thereby diminishing pain perception [8-10]. It is a simple, non-invasive, and cost-effective technique. However, evidence regarding its utility in enhancing the efficacy of IANB in SIP patients remains inconsistent. Therefore, this systematic review and meta-analysis aimed to evaluate the effectiveness of cryotherapy application in improving the anesthetic success of IANB in patients with symptomatic irreversible pulpitis.

## Review

Methods and materials

This systematic review and meta-analysis were conducted in accordance with the Preferred Reporting Items for Systematic Reviews and Meta-Analyses (PRISMA) guidelines [11] and followed the methodological recommendations outlined in the Cochrane Handbook for Systematic Reviews of Interventions [12].

Literature search

A comprehensive search strategy was employed across four electronic databases: PubMed, Web of Science (WOS), Scopus, and the Cochrane Central Register of Controlled Trials, from inception to February 2025. The search terms included: ("Cryotherapy" OR "Cryo-analgesia" OR "Precooled lidocaine" OR "Ice") AND ("Inferior Alveolar Nerve Block" OR "IANB") AND ("Symptomatic Irreversible Pulpitis" OR "SIP"). Search strategies were tailored to each database while maintaining a unified structure (Table 1). Only English-language studies conducted on humans and published after the year 2000 were included. A manual search of reference lists from all eligible studies was also performed to identify any additional relevant articles.

**Table 1:** Detailed search strategy for each database

Database	Search Terms	Search Field	Search Results
PubMed	("Cryotherapy" OR "Cryo-analgesia" OR "Precooled lidocaine" OR "Ice") AND ("Inferior Alveolar Nerve Block" OR "IANB") AND ("Symptomatic Irreversible Pulpitis" OR "SIP")	All Fields	9
Cochrane	("Cryotherapy" OR "Cryo-analgesia" OR "Precooled lidocaine" OR "Ice") AND ("Inferior Alveolar Nerve Block" OR "IANB") AND ("Symptomatic Irreversible Pulpitis" OR "SIP")	All Field	23
WOS	("Cryotherapy" OR "Cryo-analgesia" OR "Precooled lidocaine" OR "Ice") AND ("Inferior Alveolar Nerve Block" OR "IANB") AND ("Symptomatic Irreversible Pulpitis" OR "SIP")	All Field	7
Scopus	("Cryotherapy" OR "Cryo-analgesia" OR "Precooled lidocaine" OR "Ice") AND ("Inferior Alveolar Nerve Block" OR "IANB") AND ("Symptomatic Irreversible Pulpitis" OR "SIP")	Title, Abstract, Keywords	9

Eligibility criteria

Study selection followed a two-step screening process: initial title and abstract screening, followed by full-text review to identify randomized controlled trials (RCTs) that met the predefined inclusion criteria. Eligible studies met the following Population, Intervention, Comparator, Outcomes (PICO) criteria: Population (P): Patients diagnosed with symptomatic irreversible pulpitis (SIP); Intervention (I): IANB in combination with cryotherapy application; Comparator (C): IANB without cryotherapy; Outcomes (O): Success rates, pain scores (VAS), and anxiety scores (DAS-R). Only peer-reviewed RCTs reporting outcomes based on intention-to-treat analysis were included. Studies were excluded if they did not compare cryotherapy during IANB, were non-randomized, presented as conference abstracts, or involved unpublished data.

Endpoints

The primary outcome was the success rate of IANB following cryotherapy application. Secondary outcomes included pain intensity measured by the Visual Analogue Scale (VAS) and anxiety measured by the Dental Anxiety Scale-Revised (DAS-R). Both scales used a 0-10 grading system, where “0” indicated no pain or anxiety and “10” represented the worst possible pain or highest anxiety level.

Risk of bias assessment

Two independent reviewers evaluated the methodological quality of the included studies using the Cochrane Risk of Bias 2.0 (ROB 2; www.cochrane.org) tool [13]. This tool assesses five domains: randomization process, deviations from intended interventions, missing outcome data, measurement of outcomes, and selection of the reported results. Studies were categorized as having “low risk of bias,” “some concerns,” or “high risk of bias.” Disagreements were resolved through discussion with a third reviewer.

Data extraction and statistical analysis

Data extraction was conducted using a standardized Excel spreadsheet covering four domains: (1) study characteristics; (2) patient demographics; (3) risk of bias domains; and (4) outcome measures. Dichotomous data (e.g., success rates) were extracted as the number of events and total sample size, while continuous outcomes (e.g., VAS scores) were extracted as mean and standard deviation (SD) at the latest available time point.

Meta-analyses were performed using the DerSimonian-Laird random-effects model. For dichotomous outcomes, odds ratios (ORs) with 95% confidence intervals (CIs) were calculated. For continuous outcomes, mean differences (MDs) with 95% CIs were computed. Heterogeneity was assessed using Cochrane’s Q test and quantified with the I² statistic, with p < 0.05 and I² ≥ 50% indicating significant heterogeneity.

Subgroup analyses were conducted for the primary outcome based on the cryotherapy modality (Endo Ice vs. ice packs). Additionally, secondary outcomes were stratified based on the timing of assessment (preoperative, intraoperative, or one hour postoperative). All statistical analyses were conducted using STATA version 18MP (www.stata.com), utilizing the “meta esize” and “meta forest plot” packages [14-17].

Results

Search Results

**Figure 1:**
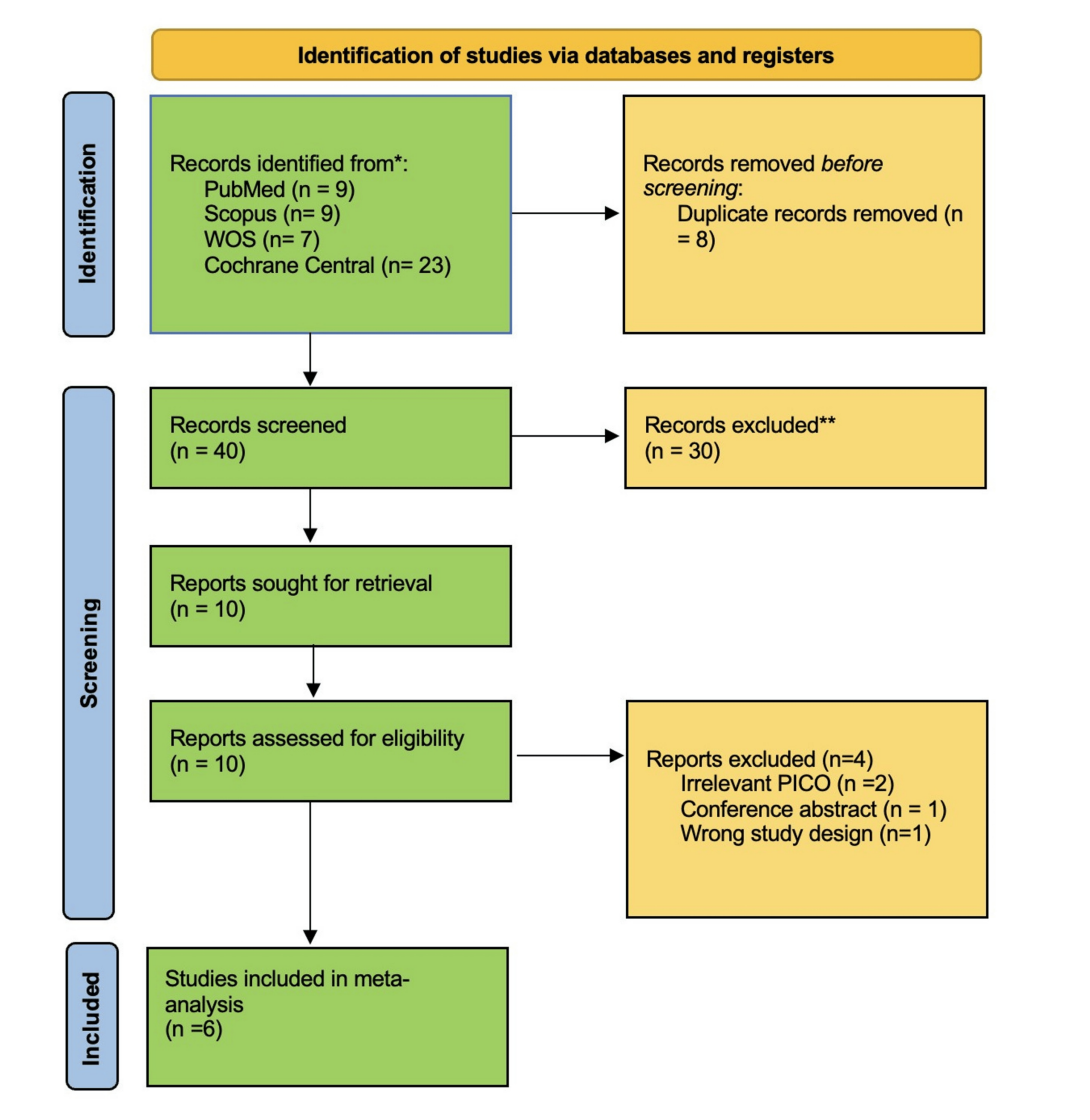
PRISMA flow chart PRISMA: Preferred Reporting Items for Systematic Reviews and Meta-Analyses.

The initial database search yielded 48 records. After the removal of duplicates and screening of titles, abstracts, and full-text articles based on the predefined PICO criteria, 42 studies were excluded. Ultimately, six RCTs [9,10,18-21] were included in the final meta-analysis. The study selection process is illustrated in the Preferred Reporting Items for Systematic Reviews and Meta-Analyses (PRISMA) flow diagram (Figure 1).

Study characteristics and risk of bias

A total of six RCTs encompassing 506 patients were included. Among them, 283 patients (55.9%) received cryotherapy as an adjunct to IANB, while 223 patients (44.1%) underwent IANB without cryotherapy. All studies followed patients until one hour postoperatively. Detailed baseline characteristics and study summaries are presented in Tables 1, 2.

**Table 2:** Summary characteristics of the included studies References: [9,10,18–21]. IANB = Inferior alveolar nerve block.

Study ID	Study Design	Sample Size	Intervention	Control	Outcomes	IANB Solution	Cryotherapy Method
Topçuoğlu et al. 2019 [9]	Randomized controlled trial	104	IANB + Cryotherapy (Ice packs)	IANB alone	Success Rate	3.6 mL 2% lidocaine with 1:100,000 epinephrine	Intraoral Ice Pack (Applied on the vestibular surface for 5 min after IANB)
Koteeswaran et al. 2018 [10]	Randomized controlled trial	40	IANB + Cryotherapy (Endo-ice)	IANB alone	Intraoperative Pain - Postoperative Pain	2 ml of 2% lignocaine hydrochloride with adrenaline 1: 80000	Endo-Ice Followed by Intrapulpal Ice Application (During pulp extirpation, after IANB)
Gopakumar et al. 2023 [18]	Randomized controlled trial	200	1- IANB + Cryotherapy (Ice Packs) 2- IANB + Cryotherapy (Endo-Ice)	IANB alone	Success Rate - Intraoperative Pain - Postoperative Pain	2 mL of 2% lignocaine hydrochloride with adrenaline 1:80000	Endo-Ice + Intrapulpal Ice Sticks (After IANB, during procedure)
Elheeny et al. 2023 [19]	Randomized controlled trial	152	IANB + Cryotherapy (Ice packs)	IANB alone	Success Rate - Intraoperative Pain - Supplementary Anesthesia - Postoperative Pain	3.6 mL of articaine hydrochloride 4%, and epinephrine, 1:100,000	Extraoral Buccal Vestibule Ice Pack (5 min after IANB)
Gupta and Prakash, 2022 [20]	Randomized controlled trial	30	1- IANB + Cryotherapy (Ice Packs) 2- IANB + Cryotherapy (Endo-Ice)	IANB alone	Success Rate - Pain Reduction	3.6 mL 2% lidocaine with 1:100,000 epinephrine	Small Ice Pack (wrapped in sterile gauze) or Endo-Ice (Before access opening, after IANB)
Karunakar et al. 2024 [21]	Randomized controlled trial	30	IANB With Ice Packs	IANB alone	Success Rate - Intraoperative Pain	1.8 mL of 2% lignocaine with 1:80,000 epinephrine	Precooled Lidocaine (4-6°C) + Intraoral Cryotherapy (After IANB)

**Table 3:** Baseline characteristics of the included patients References: [9,10,18–21]. IANB = Inferior alveolar nerve block, NA = Not available.

Study ID	Arms	Sample Size	Age		Sex (Male,n,%)
			Mean	SD	
Topçuoğlu et al., 2019 [9]	IANB + Ice Packs	52	35.81	11.35	24 (46%)
	IANB Alone	52	32.04	9.15	27 (52%)
Koteeswaran et al., 2018 [10]	IANB + Endo Ice	20	28.6	7.68	8 (40%)
	IANB Alone	20	30.8	7.38	9 (45%)
Gopakumar et al., 2023 [18]	IANB + Ice Packs	50	31	2.08	25 (50%)
	IANB + Endo Ice	50	30.8	2.11	27 (54%)
	IANB Alone	50	30.4	2.1	24 (48%)
Elheeny et al., 2023 [19]	IANB + Ice Packs	76	34.2	4.5	36 (47.4)
	IANB Alone	76	33.8	4.2	31(40.8)
Gupta and Prakash, 2022 [20]	IANB + Ice Packs	10	NA	NA	NA
	IANB + Endo Ice	10	NA	NA	NA
	IANB Alone	10	NA	NA	NA
Karunakar et al., 2024 [21]	IANB With Ice Packs	15	NA	NA	NA
	IANB Alone	15	NA	NA	NA

**Figure 2:**
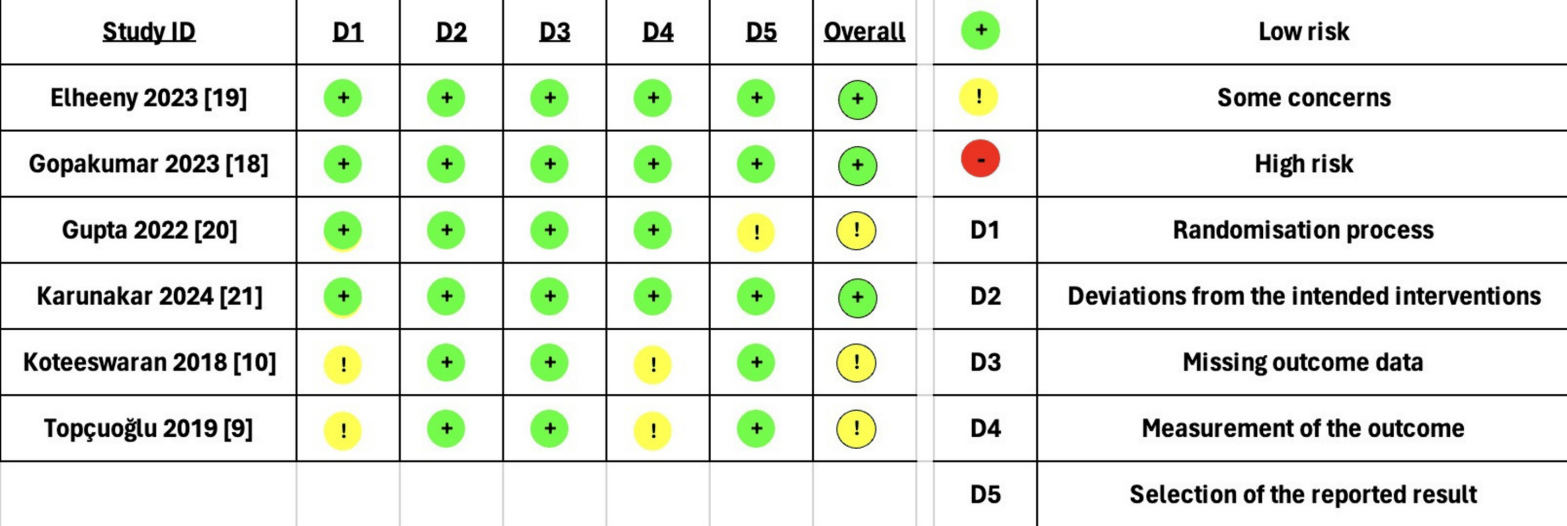
Risk of bias of the included studies References: [9,10,18–21].

Risk of bias assessment using the Cochrane ROB 2 tool revealed that three studies had a low risk of bias, whereas the remaining three were judged as having "some concerns," primarily related to issues in the randomization process and outcome measurement (Figure 2).

Primary and secondary outcomes

**Figure 3:**
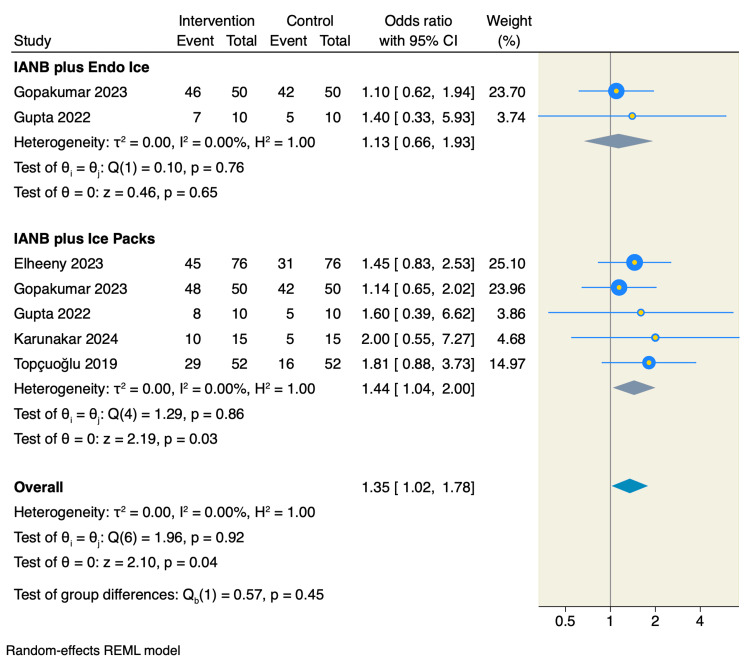
Forest plot of the success rates following IANB References: [9,10,18–21] IANB: Inferior alveolar nerve block.

Five studies reported on the success rate of IANB with and without cryotherapy. Subgroup analysis showed that the application of ice packs significantly improved the success rate compared to no cryotherapy (OR = 1.44, 95% CI: 1.04-2.00; p = 0.03; I² = 0%, p = 0.86). In contrast, Endo Ice application did not demonstrate a statistically significant difference (OR = 1.13, 95% CI: 0.66-1.93; p = 0.65; I² = 0%, p = 0.76). The overall pooled analysis across five RCTs favored cryotherapy in enhancing IANB success rates (OR = 1.35, 95% CI: 1.02-1.78; p = 0.04; I² = 0%, p = 0.92), as shown in Figure 3.

**Figure 4:**
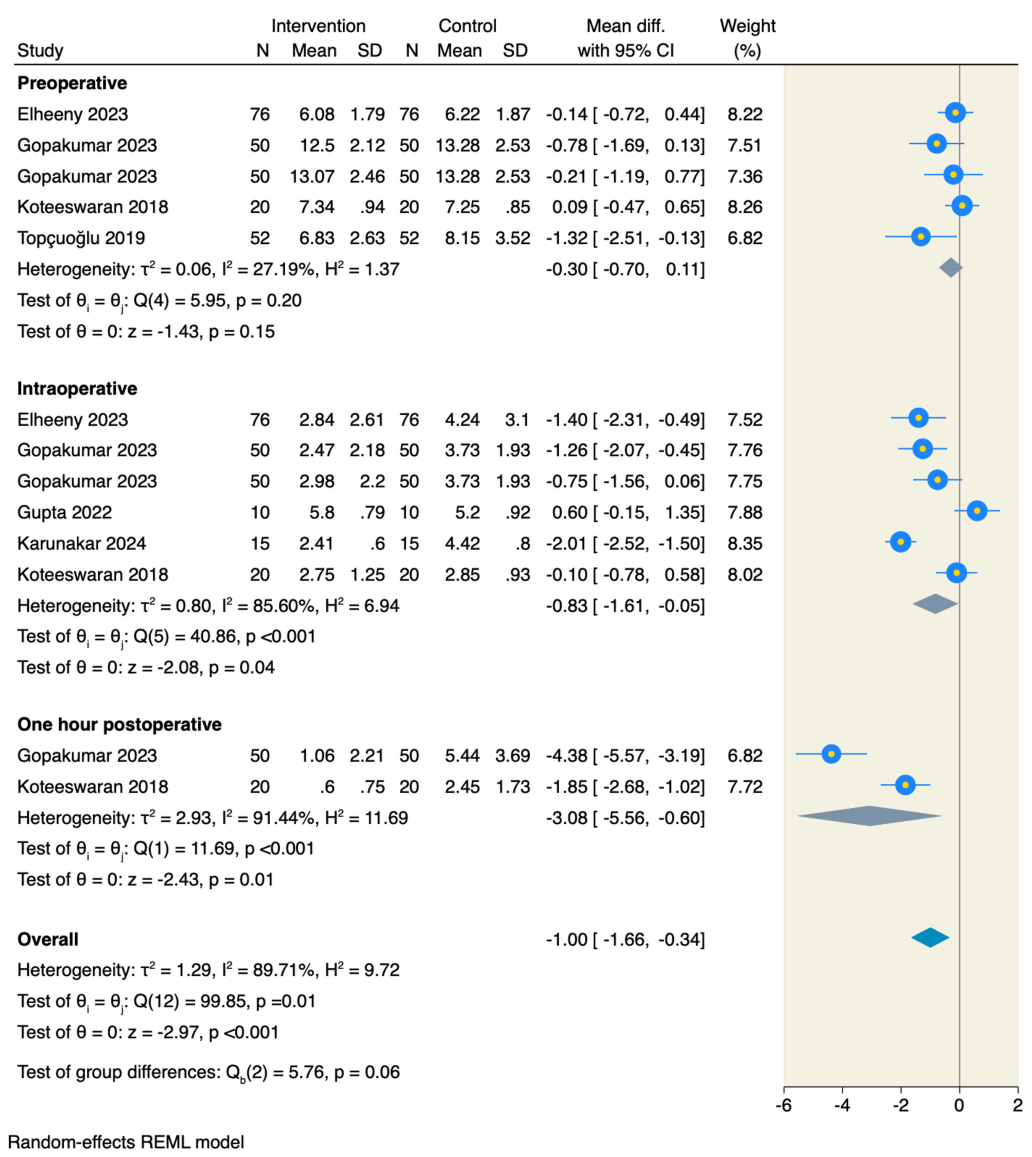
Forest plot of the pain assessment using VAS References: [9,10,18–21]. VAS: Visual analogue scale.

Cryotherapy also significantly reduced intraoperative pain measured by the Visual Analogue Scale (VAS) (MD = -0.83, 95% CI: -1.61 to -0.05; p = 0.04; I² = 85.6%, p < 0.001) and pain at one hour postoperatively (MD = -3.08, 95% CI: -5.56 to -0.60; p = 0.01; I² = 91.4%, p < 0.001). However, no significant difference was observed in preoperative pain scores between groups (MD = -0.30, 95% CI: -0.70 to 0.11; p = 0.15; I² = 27.2%, p = 0.20), as presented in Figure 4.

**Figure 5:**
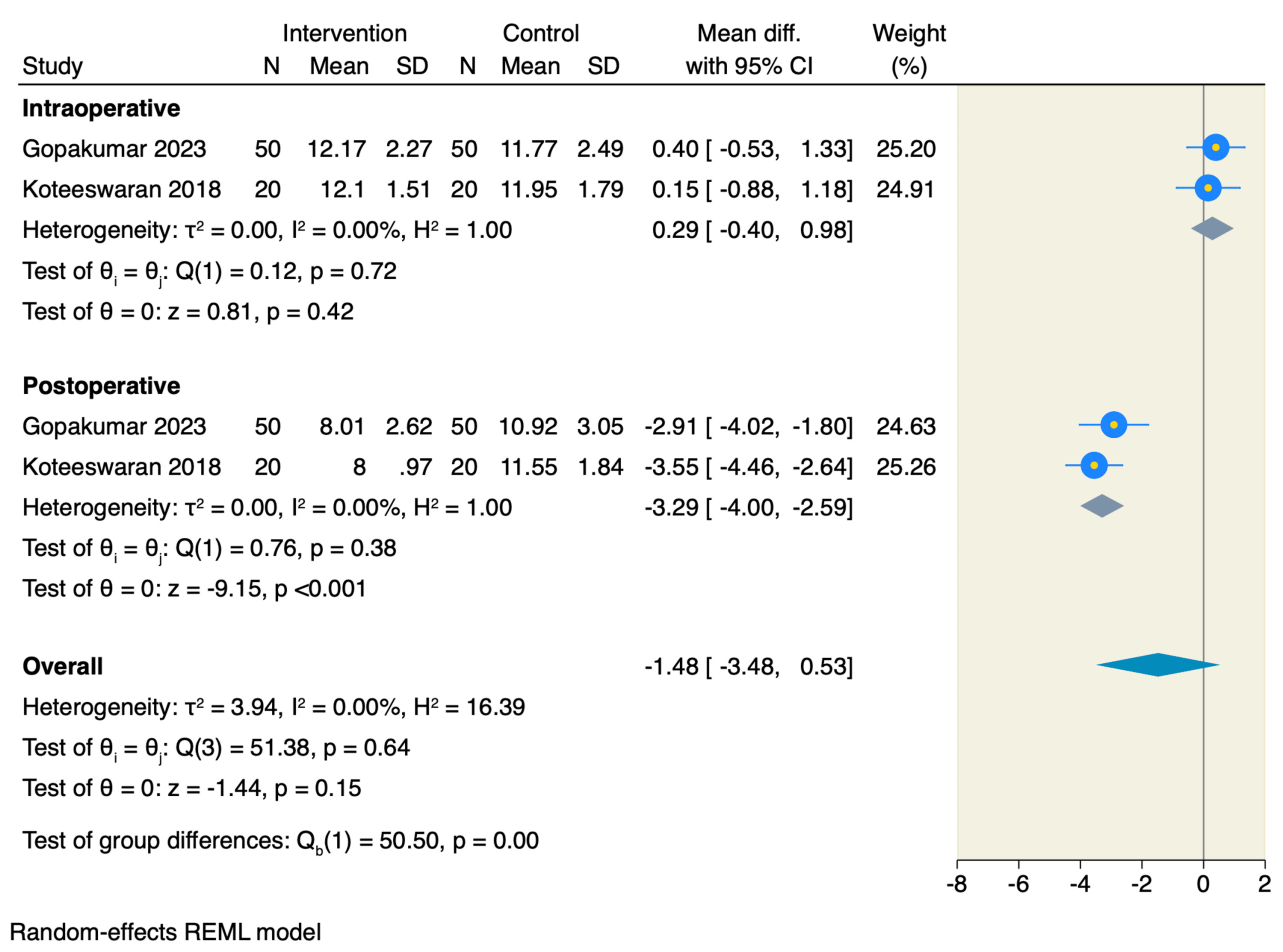
Forest plot of the dental anxiety assessment using DAS-R References: [10,18]. DAS-R: Dental Anxiety Scale-Revised.

Regarding anxiety outcomes, there was no significant difference between the two groups in intraoperative anxiety levels (MD = 0.29, 95% CI: -0.40 to 0.98; p = 0.42; I² = 0%, p = 0.72). In contrast, postoperative anxiety was significantly lower in the cryotherapy group compared to the control (MD = -3.29, 95% CI: -4.00 to -2.59; p < 0.001; I² = 0%, p = 0.38), as shown in Figure 5.

Discussion

This systematic review and meta-analysis of six randomized controlled trials involving 506 patients represents the most comprehensive synthesis to date assessing the effectiveness of cryotherapy application following inferior alveolar nerve block (IANB) in patients with symptomatic irreversible pulpitis (SIP). Our findings demonstrate that cryotherapy, particularly when delivered via ice packs, significantly enhances IANB success rates, reduces intraoperative and early postoperative pain, and lowers postoperative dental anxiety levels.

Although IANB is the standard anesthetic technique for mandibular molars, it frequently fails to achieve effective pulpal anesthesia in patients with SIP [22]. This reduced efficacy is largely attributed to the inflammatory microenvironment, wherein elevated levels of mediators such as bradykinin and prostaglandin E2 alter nerve excitability by lowering the activation threshold [23]. Because patient perceptions of endodontic treatment are closely tied to their anesthesia experience, adjunctive measures to improve IANB effectiveness are clinically valuable.

Cryotherapy has been widely applied in both acute and chronic pain management. Its analgesic effects stem from local vasoconstriction, reduced metabolic activity, and suppression of pain-related neuropeptides [9]. By lowering tissue temperature at the anesthetic site, cryotherapy impairs peripheral nerve conduction and dampens pain signal transmission [9,10]. This mechanism likely explains the observed enhancement in IANB efficacy across the included studies. Moreover, cryotherapy has the advantage of being simple, non-invasive, and cost-effective.

The majority of included studies employed a five-minute cryotherapy application window, consistent with recent recommendations to avoid soft tissue damage while optimizing analgesic effects [24]. Prolonged or overly intense thermal application may risk tissue injury, especially in areas with minimal soft tissue thickness [10]. Further age-specific and tissue-specific studies are warranted to refine optimal cryotherapy duration and technique.

Our subgroup analyses revealed that ice pack application significantly increased the success rate of IANB, while Endo Ice did not produce a statistically significant benefit. These differences may be attributed to the duration and mode of application. For example, Gupta et al. [20] found no improvement in IANB success with cryotherapy, potentially due to increased dentin thickness reducing thermal conductivity, thus limiting cooling effects [25]. Pantera et al. further emphasized that brief or inconsistent exposure to cold may be insufficient to achieve therapeutic hypothermia [26].

Additional factors influencing IANB failure include anatomical variations, accessory innervation, sodium channel resistance, and psychological responses [27,28]. Adjusting the temperature or pH of anesthetic solutions may help reduce injection pain, but cryotherapy offers a direct, localized approach to modulate pain perception. Our pooled analysis confirmed that ice pack cryotherapy reduced VAS-measured pain both intraoperatively and at one hour postoperatively, supporting its role as a valuable adjunct in managing pain during endodontic procedures.

A recent study by Karunakar et al. [21] investigating intraoral cryotherapy combined with local anesthetic reported a significant reduction in injection pain, likely due to the deactivation of A-delta fibers responsible for transmitting sharp pain sensations [29,30]. This is in line with our findings and further supports the neurophysiological basis for cryotherapy’s efficacy.

Given the well-documented correlation between preoperative and intraoperative pain [31,32], we ensured independent analyses of different time points. Additionally, included trials controlled for operator-related variables by standardizing the personnel responsible for IANB administration, cryotherapy application, and endodontic treatment. Most studies excluded patients who had taken analgesics within 12 hours preoperatively, minimizing potential confounding.

We also observed a significant reduction in postoperative dental anxiety among patients who received cryotherapy, although no difference was detected intraoperatively. Gopakumar et al. utilized the Dental Anxiety Scale-Revised (DAS-R) and reported a significant drop in postoperative anxiety in the cryotherapy group, likely reflecting enhanced comfort and improved anesthetic outcomes [18].

This study has several limitations. First, the sample sizes of the included RCTs were relatively small, potentially affecting the precision of effect estimates. However, we addressed this by pooling data across all available trials. Second, variations in individual pain thresholds may introduce heterogeneity in VAS scores, underscoring the need for future trials to stratify patients based on baseline pain levels. Third, our review focused exclusively on conventional IANB techniques; future studies should examine cryotherapy in combination with alternative anesthesia methods such as intraligamentary or intraosseous injections. Lastly, the included studies did not provide baseline data on key differences, such as the iceacks, with or without wrappers, refrigerants, or ice inserted into the pulp. Also, the size of the ice packs, their production and placement, and the thermal effect of their use are not provided adequately.

## Conclusions

The application of cryotherapy following IANB injection demonstrated higher success rates, significant reductions in both intraoperative and postoperative pain, and decreased dental anxiety levels. These findings support the potential of cryotherapy as an effective, low-cost adjunctive technique to enhance anesthetic outcomes in patients with symptomatic irreversible pulpitis. Further high-quality, large-scale clinical trials are warranted to validate these results and explore their utility in broader clinical contexts involving pain and inflammation.

## References

[1] Zanjir M, Lighvan NL, Yarascavitch C, Beyene J, Shah PS, Azarpazhooh A (2019). Efficacy and safety of pulpal anesthesia strategies during endodontic treatment of permanent mandibular molars with symptomatic irreversible pulpitis: a systematic review and network meta-analysis. J Endod.

[2] Berman LH, Hargreaves KM (2025). Cohen’s Pathways of the Pulp, 11th edition. https://shop.elsevier.com/books/cohens-pathways-of-the-pulp/berman/978-0-323-67303-7.

[3] Aggarwal V, Singla M, Rizvi A, Miglani S (2011). Comparative evaluation of local infiltration of articaine, articaine plus ketorolac, and dexamethasone on anesthetic efficacy of inferior alveolar nerve block with lidocaine in patients with irreversible pulpitis. J Endod.

[4] Monteiro MR, Groppo FC, Haiter-Neto F, Volpato MC, Almeida JF (2015). 4% articaine buccal infiltration versus 2% lidocaine inferior alveolar nerve block for emergency root canal treatment in mandibular molars with irreversible pulpits: a randomized clinical study. Int Endod J.

[5] Poorni S, Veniashok B, Senthilkumar AD, Indira R, Ramachandran S (2011). Anesthetic efficacy of four percent articaine for pulpal anesthesia by using inferior alveolar nerve block and buccal infiltration techniques in patients with irreversible pulpitis: a prospective randomized double-blind clinical trial. J Endod.

[6] Stentz D, Drum M, Reader A, Nusstein J, Fowler S, Beck M (2018). Effect of a combination of intranasal ketorolac and nitrous oxide on the success of inferior alveolar nerve block in patients with symptomatic irreversible pulpitis: a prospective, randomized, double-blind study. J Endod.

[7] Iranmanesh P, Khazaei S, Nili M, Saatchi M, Aggarwal V, Kolahi J, Khademi A (2022). Anaesthetic efficacy of incorporating different additives into lidocaine for the inferior alveolar nerve block: a systematic review with meta-analysis and trial sequential analysis. Int Endod J.

[8] Maiwand O, Makey AR (1981). Cryoanalgesia for relief of pain after thoracotomy. Br Med J (Clin Res Ed).

[9] Topçuoğlu HS, Arslan H, Topçuoğlu G, Demirbuga S (2019). The effect of cryotherapy application on the success rate of inferior alveolar nerve block in patients with symptomatic irreversible pulpitis. J Endod.

[10] Koteeswaran V, Ballal S, Natanasabapathy V, Kowsky D (2019). Efficacy of Endo-Ice followed by intrapulpal ice application as an adjunct to inferior alveolar nerve block in patients with symptomatic irreversible pulpitis-a randomized controlled trial. Clin Oral Investig.

[11] Page MJ, McKenzie JE, Bossuyt PM (2021). The PRISMA 2020 statement: an updated guideline for reporting systematic reviews. BMJ.

[12] (2025). Cochrane Handbook for Systematic Reviews of Interventions. https://training.cochrane.org/handbook.

[13] Sterne JA, Savović J, Page MJ (2019). RoB 2: a revised tool for assessing risk of bias in randomised trials. BMJ.

[14] Galbraith RF (1988). Graphical display of estimates having differing standard errors. Technometrics.

[15] Egger M, Davey Smith G, Schneider M, Minder C (1997). Bias in meta-analysis detected by a simple, graphical test. BMJ.

[16] L'Abbé KA, Detsky AS, O'Rourke K (1987). Meta-analysis in clinical research. Ann Intern Med.

[17] Everitt BS (2014). Bubble plot. Wiley StatsRef: Statistics Reference Online.

[18] Gopakumar R, Jayachandran M, Varada S, Jayaraj J, Ezhuthachan Veettil J, Nair NS (2023). Anesthetic efficacy of Endo-Ice and intrapulpal ice sticks after inferior alveolar nerve block in symptomatic irreversible pulpitis: a randomized controlled study. Cureus.

[19] Elheeny AA, Sermani DI, Saliab EA, Turky M (2023). Cryotherapy and pain intensity during endodontic treatment of mandibular first permanent molars with symptomatic irreversible pulpitis: a randomized controlled trial. Clin Oral Investig.

[20] Gupta R, Prakash P (2022). Cryotherapy as an adjunct to inferior alveolar nerve block in symptomatic irreversible pulpitis: a randomised controlled clinical trial. Indian J Conserv Endod.

[21] Karunakar P, Solomon RV, Kumar BS, Reddy SS (2024). Evaluating the pain at site, onset of action, duration and anesthetic efficacy of conventional, buffered lidocaine, and precooled lidocaine with intraoral cryotherapy application in patients with symptomatic irreversible pulpitis: a clinical study. J Conserv Dent Endod.

[22] Gade V, Barfiwala D, Asani R, Gawande R, Gade J (2020). Cryotherapy: an emerging trend in the field of endodontics. Int J Drug Res Dent Sci.

[23] Petrini M, Ferrante M, Ciavarelli L, Brunetti L, Vacca M, Spoto G (2012). Prostaglandin E2 to diagnose between reversible and irreversible pulpitis. Int J Immunopathol Pharmacol.

[24] Vera J, Ochoa J, Romero M (2018). Intracanal cryotherapy reduces postoperative pain in teeth with symptomatic apical periodontitis: a randomized multicenter clinical trial. J Endod.

[25] de Morais CA, Bernardineli N, Lima WM, Cupertino RR, Guerisoli DM (2008). Evaluation of the temperature of different refrigerant sprays used as a pulpal test. Aust Endod J.

[26] Pantera EA, Anderson RW, Pantera CT (1993). Reliability of electric pulp testing after pulpal testing with dichlorodifluoromethane. J Endod.

[27] Kattan S, Lee SM, Hersh EV, Karabucak B (2019). Do buffered local anesthetics provide more successful anesthesia than nonbuffered solutions in patients with pulpally involved teeth requiring dental therapy?: A systematic review. J Am Dent Assoc.

[28] Hawker GA, Mian S, Kendzerska T, French M (2011). Measures of adult pain: Visual Analog Scale for Pain (VAS Pain), Numeric Rating Scale for Pain (NRS Pain), McGill Pain Questionnaire (MPQ), Short-Form McGill Pain Questionnaire (SF-MPQ), Chronic Pain Grade Scale (CPGS), Short Form-36 Bodily Pain Scale (SF-36 BPS), and Measure of Intermittent and Constant Osteoarthritis Pain (ICOAP). Arthritis Care Res (Hoboken).

[29] Mohiuddin I, Setty JV, Srinivasan I, Desai JA (2015). Topical application of local anaesthetic gel vs ice in pediatric patients for infiltration anesthesia. J Evol Med Dent Sci.

[30] Gurucharan I, Sekar M, Balasubramanian S, Narasimhan S (2022). Effect of precooling injection site and cold anesthetic administration on injection pain, onset, and anesthetic efficacy in maxillary molars with symptomatic irreversible pulpitis: a randomized controlled trial. Clin Oral Investig.

[31] Kayaoglu G, Gürel M, Saricam E, Ilhan MN, Ilk O (2016). Predictive model of intraoperative pain during endodontic treatment: prospective observational clinical study. J Endod.

[32] Martín-González J, Echevarría-Pérez M, Sánchez-Domínguez B, Tarilonte-Delgado ML, Castellanos-Cosano L, López-Frías FJ, Segura-Egea JJ (2012). Influence of root canal instrumentation and obturation techniques on intra-operative pain during endodontic therapy. Med Oral Patol Oral Cir Bucal.

